# Supplementary material for: Parents’ perceptions of core outcomes in neonatal research in two Nigerian neonatal units
Source: BMJ Paediatr Open. 2020 Jun 4;4(1):e000669. doi: 10.1136/bmjpo-2020-000669 (PMC7279647; doi:10.1136/bmjpo-2020-000669)
Supplement: Supplementary data [file bmjpo-2020-000669supp001.pdf]

SUPPLEMENTARY MATERIAL

Supplement 1: Interview guide for parents

Can you describe what has happened during the time you and your baby have spent on the neonatal unit/KMC?

If necessary, prompt

Good things about their time on the unit

Difficult things about their time on the unit

Do you have any concerns for your baby's and your family's future? If yes, what are these concerns?

If necessary, prompt

Health concerns

Financial concerns

Education concerns

What is important to you for your baby's future?

Define an outcome e.g. an outcome is something researchers measure to look at the effect's treatments have on newborn babies in order to help doctors and other health professionals make decisions about how to treat these babies.

What outcomes do you think are important to measure to see if your baby's care has been effective?

If necessary, prompt

Feeding e.g. time to breastfeeding

Complications e.g. new diagnoses

Neonatal unit vs KMC

What were your expectations of treatment/treatment outcomes?

Have your expectations changed during your time on the unit/KMCs
